# Draft Genome Sequence and Annotation of the Phytopathogenic Ralstonia pickettii (Previously Burkholderia glumae) Strain ICMP-8657

**DOI:** 10.1128/genomeA.00128-18

**Published:** 2018-03-08

**Authors:** Julia Paterson, Harald Gross

**Affiliations:** a Department of Pharmaceutical Biology, Institute of Pharmaceutical Sciences, University of Tübingen, Tübingen, Germany; b German Centre for Infection Research (DZIF), Partner Site Tübingen, Tübingen, Germany

## Abstract

Strain ICMP-8657 was formerly taxonomically classified as Burkholderia glumae and reported to be the producer of an antibacterial pyrazole derivative. Here, we report the draft genome sequence of ICMP-8657, which failed to demonstrate the biosynthetic capacity to produce the stated antibacterial compound, leading to its taxonomic reclassification as Ralstonia pickettii ICMP-8657.

## GENOME ANNOUNCEMENT

The plant-pathogenic strain ICMP-8657 was originally isolated from infected rice seedlings in Yatabe, Japan, and described as Pseudomonas glumae. In 1994, P. glumae was transferred to the genus Burkholderia as B. glumae (1, 2). In 2008, Mitchell and coworkers reported the isolation of a structurally unusual pyrazole compound, which exhibited marked antibacterial activity toward Erwinia amylovora, from B. glumae strains ICMP-3729 and ICMP-8657 (3). In order to identify the corresponding biosynthetic gene cluster (BGC) and to facilitate biosynthetic studies, we obtained the strains from the International Collection of Microorganisms from Plants (ICMP), New Zealand, and sequenced their genomes. Strain ICMP-3729 was found to be a true B. glumae strain, while the taxonomy of strain ICMP-8657 required revision. This work presents the genome sequence of strain ICMP-8657, a genome-based taxonomic revision, and annotation analyses uncovering its secondary metabolism and resistance genes.

The genome of ICMP-8657 was sequenced using a combined Illumina/PacBio sequencing approach. Genomic DNA (gDNA) was first subjected to 125-cycle paired-end sequencing with the Illumina HiSeq 2500 system. The first *de novo* assembly was performed using the CLC Genomics Workbench 7.0.4, yielding 2,780 contigs. To improve the quality of the sequence, the genome was resequenced using the PacBio RS II technology (10-kb library, 239,325 reads, 1,233 Mb, and 4.205 bp average read length). The quality of the Illumina reads was improved by trimming off low-quality bases using BBDuk (BBMap suite version 36.77). High-quality reads were assembled into contigs using ABySS version 2.0.2 (4). The long reads were mapped to the draft assembly using Basic Local Alignment with Successive Refinement (BLASR) (5). Based on these alignments, the contigs were linked and placed into scaffolds. The orientation, order, and distance between the contigs were estimated using SSPACE-LongRead version 1.0 (6). Using Illumina reads, gapped regions within scaffolds were (partially) closed using GapFiller version 1.10 (7). Finally, assembly errors and nucleotide disagreements between the Illumina reads and scaffold sequences were corrected using Pilon version 1.21 (8).

The final draft genome of ICMP-8657 generated 72 contigs, with a sequence length of 5,484,397 bp (63.7% G+C content), which is consistent with other sequenced R. pickettii genomes (4.8 to 8.1 Mb and 63.3 to 65.0% G+C content) (9–13).

Extraction of the 16S rRNA using RNAmmer (14) and subsequent BLAST comparision revealed that the 16S rRNA of strain ICMP-8657 is 100% identical to that of Ralstonia pickettii 12D (GenBank accession number NC_012856). The transfer of ICMP-8657 to the genus Ralstonia is not unexpected, since several Burkholderia and Alcaligenes species were previously transferred to the genus Ralstonia (15). R. pickettii strains are mainly known to cause nosocomial human infections (16, 17), but beyond clinical settings, they can be isolated from water and soil (18–23), as with the collection site of ICMP-8657. Using antiSMASH 4.0.2 (24), only 4 BGCs coding for putative secondary metabolites were predicted: a bacteriocin, an aryl polyene (25), a terpenoid, and a citrate-based, most likely a staphyloferrin-B/schizokinen/rhizoferrin-like, siderophore, consistent with other R. pickettii strains (26–29). The presence of antimicrobial resistance genes was inferred based on ResFinder 3.0 (30) and manual BLAST searches; the sequences showed significant identity with the beta-lactamase genes *bla*_OXA-22_ (100.0%), *bla*_OXA-60_ (100%), and VanZ (100%).

### Accession number(s).

This whole-genome shotgun project has been deposited at DDBJ/ENA/GenBank under the accession number NREQ00000000. The version described in this paper is version NREQ02000000.
